# Comparision of profile macro-estethic perception among orthodontists, dentistry students, orthodontic patients and surgical orthodontic patients

**DOI:** 10.4317/jced.57593

**Published:** 2020-12-01

**Authors:** Michele Cassetta, Rosanna Guarnieri, Martina Mezio, Federica Altieri, Giulia Brandetti, Gabriella Padalino, Roberto Di Giorgio, Ersilia Barbato

**Affiliations:** 1 Associated Professor, DDS, PhD, Department of Oral and Maxillofacial Sciences, School of Dentistry, “Sapienza” University of Rome, Italy; 2 Research Assistant, DDS, PhD, Department of Oral and Maxillofacial Sciences, School of Dentistry, “Sapienza” University of Rome, Italy; 3 DDS, School of Orthodontics, “Sapienza” University of Rome, Italy; 4 Research Assistant, DDS, PhD Student, Department of Oral and Maxillofacial Sciences, School of Dentistry, “Sapienza” University of Rome, Italy; 5 MS, Statistician; 6 Associated Professor, MD, DDS, PhD, Department of Oral and Maxillofacial Sciences, School of Dentistry, “Sapienza” University of Rome, Italy; 7 Full Professor and Department Chair, DDS, PhD, Department of Oral and Maxillofacial Sciences, School of Dentistry, “Sapienza” University of Rome, Italy

## Abstract

**Background:**

The patient’s needs should guide the orthodontist in choosing the most appropriate therapy. The purpose of the present survey was to compare the esthetic perception of the facial profile by orthodontists (O), dentistry students (DS), orthodontic patients (OP) and surgical-orthodontic patients (SOP) and to evaluate the influence of gender, age and level of study.

**Material and Methods:**

A facial profile photograph of a young female was taken and twelve modified images were made, altering the position of the jaws in protrusion and in retrusion. Two hundred caucasian examiners, divided into four groups (O, DS, OP, SOP), were selected. Each examiner was asked to complete the questionnaire with an approval rating from 1 to 10. An ordinary least square OLS model was applied. Significant levels were set at *P* ≤ 0.05.

**Results:**

All examiners considered a straight profile or a slight retrusion of the maxilla as the most attractive profile. Slight discrepancies (up to 2 mm) in jaw protrusion were barely perceived by patients. Mandibular retrusion (2 and 4 mm) was one of the least appreciated condition by all examiners. Surgical-orthodontic patients assigned lower ratings compared to orthodontic patients. Female subjects assigned lower ratings than males. Patients with secondary school education assigned higher statistically significant values compared to other levels of study. The lowest values were attributed by the sample of age > = 17 years.

**Conclusions:**

The choice of the most appropriate therapy is based not only on a correct diagnosis, but on the evaluation of esthetic and psychological aspects.

** Key words:**Estethic, profile, orthodontic, surgical orthodontic patients.

## Introduction

The evaluation of facial esthetics in orthodontic clinical examination is an important step to meeting the patient’s needs (1). Evaluating the patient’s esthetics requires a combined analysis of face and smile, and it should not be limited to the evaluation of the individual components. Over the years, treatment planning has changed from an occlusal evaluation based on cephalometric standards, to an esthetic evaluation based on soft-tissue analyses (2,3). Several studies in the literature have demonstrated the absence of correlation between the achievement of cephalometric and occlusal standards at the end of treatment and the patient’s satisfaction in terms of esthetic result (4). In the last decade, the world of social networks and the “selfie generation” (5) has emphasised the centrality of face esthetics even among adolescents, who are increasingly aware of their physical appearance. Over the years numerous studies in literature have tried to define the gold standard of facial esthetics; some studies have analyzed the differences in esthetic perception existing between orthodontists and laypersons (6-8,9) between orthodontists and orthodontic patients (10,11), between children with and without orthodontic history (12) and between different ethnic groups (13). No study in the literature has analyzed the differences in esthetic perception between different categories of patients. In the evaluation of macroesthetics, several studies have analyzed the perception of the facial profile (14). The variability of esthetic standards over the years (2) introduces the concept of “dynamic facial esthetics”. The analysis of the modern model of facial esthetics should be based only on the most recent studies (within the last 10 years), at the same time taking into account the need to periodically consider new models (15).

The purpose and primary outcome of the present survey was to compare the esthetic perception of the facial profile of a young female by orthodontists (O), dentistry students (DS), orthodontic patients (OP) and surgical-orthodontic patient (SOP) and to evaluate the influence of gender, age and level of study as a secondary outcome. The Authors hypothesized that there is not a different facial profile perception between the defined populations and that this perception is not influenced by the investigated variables.

## Material and Methods

Subject choice and original photo capture 

**Figure 1:**
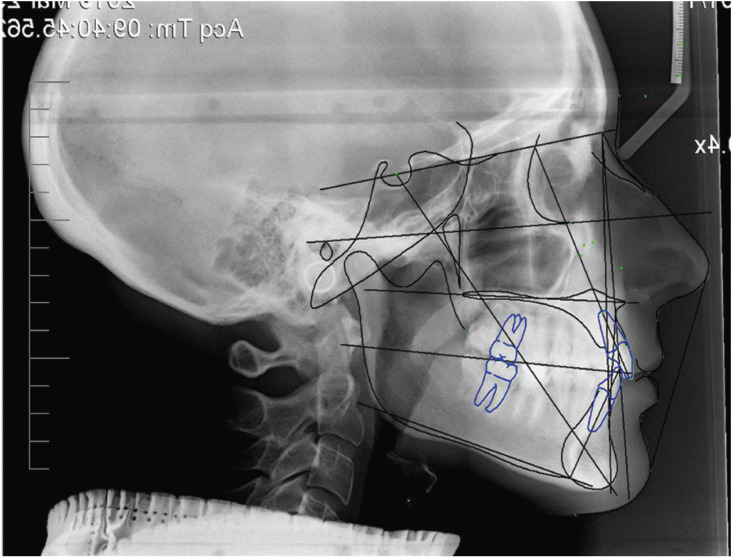
Cephalometric analysis of soft and hard tissues.

A 23-year-old female was selected. Considering the soft and hard tissues cephalometric analysis, esthetic and skeletal parameters were within the normal range (Fig. 1, Table 1).

**Table 1:**
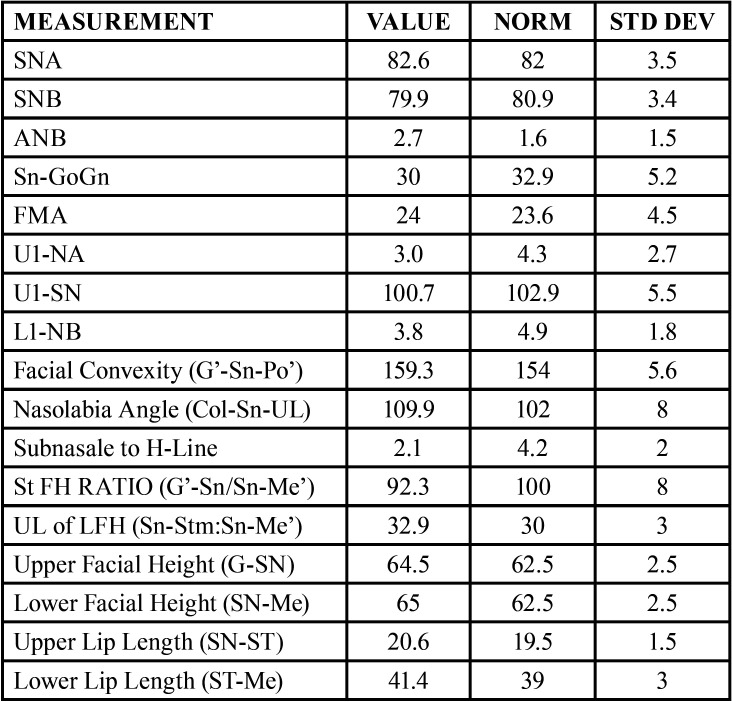
Cephalometric analysis values.

A photograph of the subject’s profile was taken in natural head posture with relaxed position of the lips (Nikon AF-S 28-300 mm, f/3.5-5.6, VR, EU version). The camera was placed at the same height as the face, at right angles. The subject was asked to stand and look forward with relaxed arms at her sides. The photo was taken in colour (Figs. 1,3).

**Figure 2:**
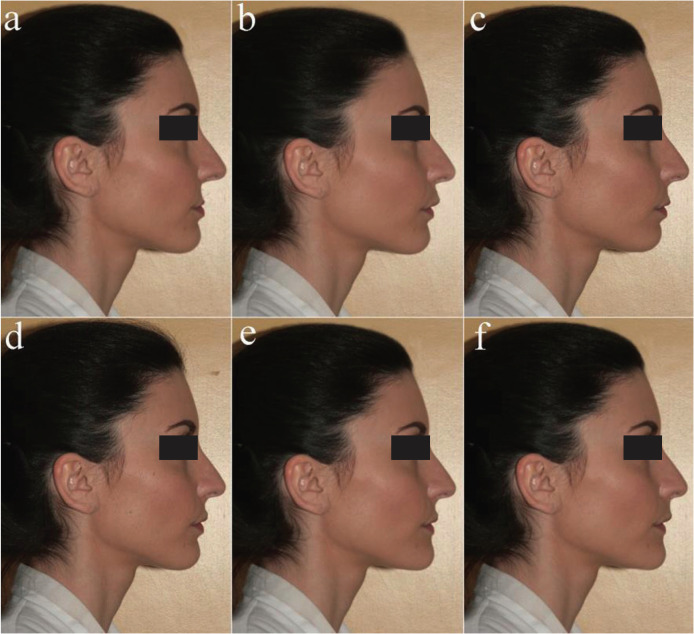
a) Retrognathic maxilla of 4 mm; b) Prognathic maxilla of 2 mm; c) Bimaxillary dentoalveolar retrusion of 2 mm; d) Original photography (straight profile); e) Prognathic mandible of 2 mm; f) Bimaxillary dentoalveolar protrusion of 2 mm.

**Figure 3:**
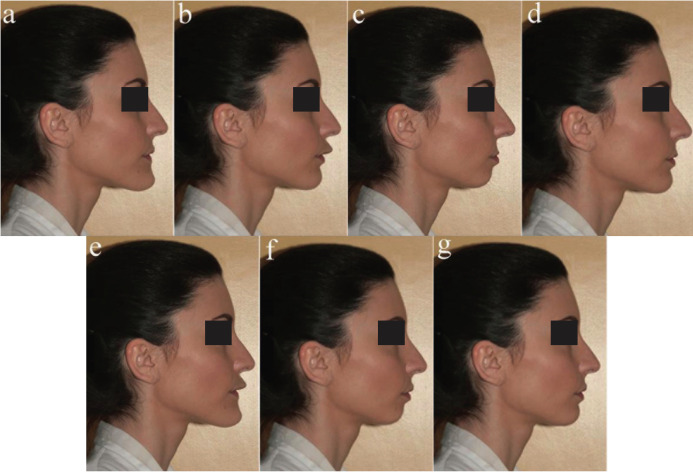
a) Prognathic mandible of 4 mm; b) Prognathic maxilla of 4 mm; c) Bimaxillary dentoalveolar retrusion of 4 mm; d) Retrognathic maxilla of 2 mm; e) Bimaxillary dentoalveolar protrusion of 4 mm; f) Retrognathic mandible of 4 mm; g) Retrognathic mandible of 2 mm.

*Pi*cture manipulation

The original picture was modified using software (Photoshop, Adobe System, San Jose, Calif.). Twelve modified images of the profile, standardized in size, were made, altering the position of the jaws in protrusion and in retrusion (Figs. 2,3). The twelve modified images and the original photo were randomly arranged in order to create a questionnaire.

-Sample selection

Two-hundred Caucasian examiners were selected at the Orthognathic Unit of the Policlinico Umberto I and at Sapienza University of Rome and divided in four groups: orthodontists (O); students in the last year of their degree in Dentistry (DS); orthodontic patients (OP); surgical-orthodontic patients (SOP) who were undergoing pre-surgical orthodontic treatment.

-Questionnaire 

The present investigation was undertaken after informing the examiners or the relative parents/guardians of the content of the study and after obtaining written consent. All examiners have been instructed in how to fill in the questionnaire, and informed that participation was voluntary. No information was provided to the examiners regarding the purposes of the survey and the changes made to the original photograph. The questionnaire also collected the following personal data: a) age (>17 years; <17 years); b) gender (M;F); c) level of study (primary school, secondary school, high school, university). Each examiner was asked to complete the questionnaire with an approval rating from 1 (extremely unattractive) to 10 (extremely attractive) for each of the 13 images through the Visual Analog Scale (VAS). All images with a score above 6 were considered to satisfy the examiner.

-Statistical analysis

Descriptive statistics were used and an ordinary least square (OLS) model was applied in order to evaluate the influence of the following categories on the variable profile: a) examiner [orthodontist (O), dentistry student (DS), orthodontic patient (OP) and surgical-orthodontic patient (SOP)]; b) gender (male: M ; female: F ); c) age (<17years; >17years); d) level of study (primary school, secondary school, high school, university). The demographic characteristics of the study sample are described in Table 2. The mean values assigned by all examiners were considered and the differences between the four categories (O, DS, OP, SOP) (Table 3), among gender (F-M) (Table 4) and age (<17years; >17years) (Table 5) were determined.

**Table 2:**
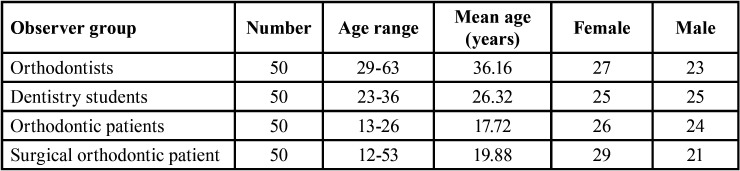
Distribution of examiner categories by gender and age.

**Table 3:**
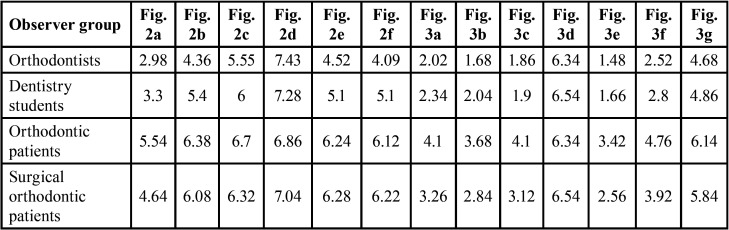
Distribution of average values considering the examiner categories.

**Table 4:**
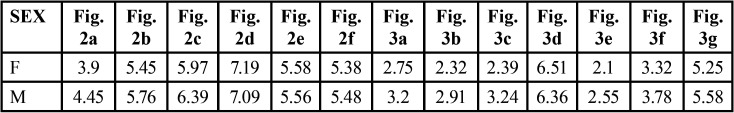
Distribution of average values considering gender.

**Table 5:**
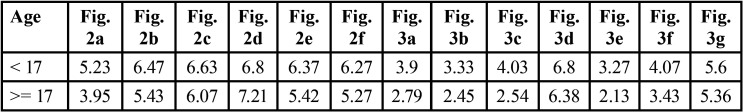
Distribution of average values considering age.

In order to obtain more detailed information about the different aesthetic perceptions among patients (OP; SOP) and experts (O; DS), a further statistical analysis was carried out. The average values assigned by the two different types of patients, OP and SOP, were investigated and compared (Table 3). The influence of gender (Table 6), age, and study levels (primary school, secondary school, high school, university) (Table 7) was also investigated. The mean value assigned by orthodontist (O) and dentistry student (DS) were finally compared (Table 3) and the influence of gender evaluated (Table 8). Significant levels were set at *P* ≤ 0.05.

**Table 6:**
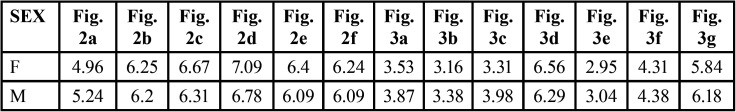
Distribution of average values considering gender in the patient groups (OP; SOP).

**Table 7:**
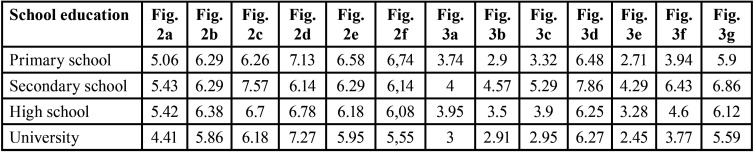
Distribution of average values considering study level in patient groups (OP; SOP).

**Table 8:**
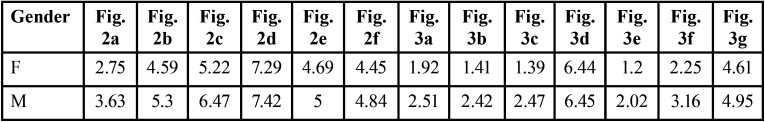
Distribution of average values considering gender in expert groups (O; DS).

## Results

All the examiners correctly completed the questionnaires, which were subsequently statistically analysed. The demographic characteristics of the study sample are described in Table 2. Considering the influence of examiner categories, the only two images that were assigned satisfactory scores by all four examiner groups were the original photo with straight profile (Fig. 2d) (average value: 7.15) and the photo with maxillary retrusion of 2 mm (Fig. 3d) (average value: 6.44). Bimaxillary dentoalveolar retrusion of 2 mm (Fig. 2c) was considered satisfactory by all groups except orthodontists (*p*=0.050). Prognathic maxilla and prognathic mandible of 2 mm were assigned satisfactory scores only by patients (OP; SOP): prognathic maxilla of 2 mm (Fig. 2b) (*p*=0.000), prognathic mandible of 2 mm (Fig. 2e) (*p*=0.000), bimaxillary dentoalveolar protrusion of 2 mm (Fig. 2f) (*p* = 0.000). Retrognathic mandible of 2 mm (Fig. 3g) was assigned a satisfactory score only by orthodontic patients (OP), (average value: 6.44), (*p*=0.000). Retrognathic mandible and retrognathic maxilla of 4 mm (Figs. 3f,2a), bimaxillary dentoalveolar protrusion and retrusion of 4 mm (Fig. 3e,c) were considered unsatisfactory by all observer groups; orthodontists (O) and dentistry students (DS) assigned lower scores, with statistically significant differences compared to the other two groups (*p*=0.000). Table 3 shows the average values attributed to each image by the four groups of examiners. Female examiners attributed lower scores to all images compared to male counterparts, with statistically significant differences in the cases of retrognathic maxilla of 4 mm (*p*=0.030) (Fig. 2a), prognathic maxilla of 4 mm (*p*=0,012) (Fig. 3b), prognathic mandible of 4 mm (*p*=0.049) (Fig. 3a), bimaxillary dentoalveolar retrusion (*p*=0.000) and bimaxillary dentoalveolar protrusion of 4 mm (*p*=0.028) (Fig. 3c,e) (Table 4). Considering the distribution of average values by age (Table 5), it was observed that the group of examiners aged> 17 years expressed lower values.

Evaluating the average scores (Table 3) assigned by the two types of patient examiner (OP, SOP), surgical-orthodontic patients attributed lower scores to all images as compared to orthodontic patients. Statistically significant differences were observed in retrognathic maxilla of 4 mm (*p*=0.006) (Fig. 2a), prognathic maxilla of 4 mm (*p*=0.003) (Fig. 3b), prognathic mandible of 4 mm (*p*=0.007) (Fig. 3a), retrognathic mandible of 4 mm (*p* = 0.000) (Fig. 3f), bimaxillary dentoalveolar retrusion of 4 mm (*p*=0.000) (Fig. 3c), bimaxillary dentoalveolar protrusion of 4 mm (*p*=0.002) (Fig. 3e). Evaluating the influence of gender, female examiners attributed lower scores to all images compared to males, with statistically significant difference in the cases of bimaxillary dentoalveolar retrusion of 4 mm (*p*=0.018) (Fig. 3c) (Table 6). The patients with secondary school level education assigned higher statistically significant values compared to other education levels (Table 7).

Evaluating the two expert groups [O; DS], orthodontists (O) assigned lower scores when compared to dentistry students (DS), with statistically significant differences in the cases of prognathic maxilla of 2 mm (*p*=0.027) (Fig. 2b) and bimaxillary dentoalveolar protrusion of 2 mm (*p*=0.008) (Fig. 2f). Female subjects attributed lower scores to all images than males (Table 8) with statistically significant difference in the cases of retrognathic maxilla of 4 mm (*p*=0.015) (Fig. 2a), prognathic mandible of 4 mm (*p*=0.044) (Fig. 3a), bimaxillary dentoalveolar retrusion of 4 mm (*p*=0.000) (Fig. 3c), bimaxillary dentoalveolar protrusion of 4 mm (*p*=0,000) (Fig. 3e), retrognathic mandible of 4 mm (*p*=0.009) (Fig. 3f).

## Discussion

The hypothesis of the study is rejected as there was a different facial profile perception between the studied populations, and this perception is influenced by the investigated variables. The original image utilized in the present study represents a dentoskeletal Class I malocclusion with harmony of the skeletal and aesthetic parameters, evaluated on lateral cephalometric radiographs and on lateral photos (Figs. 1,2d). A 23-year-old female was selected in order to make the evaluation of the esthetic parameter of the profile as realistic as possible, this differs from other studies which employ a silhouette (9,11).

All examiners considered the straight profile (Fig. 2d) or a slight retrusion of the maxilla (Fig. 3d) as the most attractive profile. This result can be interpreted in line with international literature, according to which straight profiles are generally preferred while convex profiles are considered of low esthetic appeal (9,12). In line with the results of this study, international literature reports several studies in which a Class III profile is considered the most attractive (14,16-18). However, the preference of concave profiles with prominent chin is expressed by males more than females (14). In the present study the preference for Class III profiles is not as strong, an explanation of which may be that the model is a female. The next most appreciated image is the bimaxillary dentoalveolar retrusion of 2 mm (Fig. 2c), judged as satisfactory by all groups, except orthodontists. The data is in agreement with other results observed in the literature (9,19). The preference of a bimaxillary retrusion profile could be due to the greater prevalence of this profile in the Italian population and in the Caucasian race. Prognathic maxilla and prognathic mandible of 2 mm have been assigned satisfactory scores only by patients. This finding shows that slight discrepancies (up to 2 mm) in jaw protrusion may not be perceived by patients whereas such discrepancies are perceived as unsatisfactory by orthodontists and dentistry students. On the other hand, severe discrepancies (4mm), both in protrusion and in retrusion, are negatively perceived by all examiners. The mandibular retrusion (2 mm and 4 mm) is one of the least appreciated condition, even among patients. The low rating assigned to convex profiles could be due to the influence of mass media, which propose models of beauty with evident mandibular protrusion. The position of the chin has a strong impact on the valuation of facial harmony, to the point that genioplasty has become a routine practice for the esthetic correction in the lower third of the face (20). In borderline cases, therefore, a careful esthetic and not just cephalometric evaluation becomes essential for a conscious choice between an orthodontic treatment with camouflage and an orthodontic-surgical treatment (12).

The studies currently available in the literature on this topic do not analyze the different facial profile perception between the different types of patients. This study has analyzed the differences in esthetic preferences among two types of patients: orthodontic and surgical-orthodontic. With regard to the latter group, the main reasons for surgical-orthodontic treatment are improvements in self-perception and oral function (21). The patient is less satisfied with the final result when he or she is driven by functional rather than esthetic reasons. It is important for the clinician to consider the pre-treatment reason as this may affect the patient’s final degree of satisfaction. The present results show that there is a greater awareness in the evaluation of the macroesthetic parameter of profile in the surgical-orthodontic patients. They expressed more negative judgments compared to orthodontic patients in the evaluation of the macroesthetic parameter of profile (Table 3). The judgement becomes more negative and statistically significant in the case of severe discrepancies of the jaws of 4 mm. As the clinician has the task of making a complete diagnosis that takes into account not only the cephalometric parameter, but also the esthetic and psychological one, the psychological profile of the surgical-orthodontic patient should be assessed to ascertain the possible existence of psychological problems such as appearance-related depression and anxiety (22).

Orthodontists and dentistry students show comparable results, with statistically significant differences in the facial profile perception when a 2 mm protrusion discrepancies is considered. These results indicate that postgraduate training in orthodontics allows examiners to perceive slight changes (up to 2 mm) in the facial profile. Female subjects express more negative judgements than male, which may be due to factors such as greater social conditioning as regards the evaluation of physical aesthetics. These issues have not been analyzed in the relevant literature yet. It would be advisable to carry out the same assessments also with a male model. Patients with secondary school level education assign higher statistically significant values compared to other study levels (Table 7). The data disaggregated by age group show that the lowest values are attributed by the sample of age > = 17 years (Table 5). This finding reflects the greater esthetic awareness in the post-adolescent age as compared to adolescent patients (secondary school education), who are often encouraged by their parents to start orthodontic treatment.

## Conclusions

• All examiners consider straight profile or a slight retrusion of the maxilla as the most attractive profile. Bimaxillary dentoalveolar retrusion of 2 mm was judged as satisfactory by all groups, except orthodontists;

• Slight discrepancies (up to 2 mm) in jaw protrusion are barely perceived by patients, while severe discrepancies (4mm), both in protrusion and in retrusion, are negatively perceived by all examiners:

• Mandibular retrusion (2 and 4 mm) is one of the least appreciated condition;

• The surgical-orthodontic patients express more negative judgements compared to orthodontic patients. Judgements become more negative and statistically significant in the case of severe discrepancies of the jaws of 4 mm;

• Female subjects give more negative judgements than male;

• Patients with secondary school study level assign higher statistically significant scores compared to patients with other study levels;

• The sample of age > = 17 years assigned the lowest scores;

• The clinician has the task of making a complete diagnosis that takes into account not only the cephalometric parameter, but also the esthetic and psychological one.
